# Isolation and Genomic Analysis of *Escherichia coli* Phage AUBRB02: Implications for Phage Therapy in Lebanon

**DOI:** 10.3390/antibiotics14050458

**Published:** 2025-04-30

**Authors:** Tasnime A. Abdo Ahmad, Samar A. El Houjeiry, Antoine Abou Fayad, Souha S. Kanj, Ghassan M. Matar, Esber S. Saba

**Affiliations:** 1 Department of Experimental Pathology, Immunology, and Microbiology, Faculty of Medicine, Center for Infectious Diseases Research, American University of Beirut, Beirut 1107 2020, Lebanon; ta89@aub.edu.lb (T.A.A.A.); sae62@mail.aub.edu (S.A.E.H.); aa328@aub.edu.lb (A.A.F.); gmatar@aub.edu.lb (G.M.M.); 2 Division of Infectious Diseases, Department of Internal Medicine, Center for Infectious Diseases Research, American University of Beirut Medical Center, Beirut 1107 2020, Lebanon; sk11@aub.edu.lb

**Keywords:** *Escherichia coli*, phage, lytic, biofilm, antibacterial resistance

## Abstract

Background/Objectives: *Escherichia coli* (*E. coli*), a prevalent Gram-negative bacterium, is a frequent cause of illness. The extensive use of antibiotics has led to the emergence of resistant strains, complicating antimicrobial therapy and emphasizing the need for natural alternatives such as phages. Methods: In this study, a novel *Escherichia coli* phage, AUBRB02, was isolated from sewage and characterized through whole-genome sequencing, host range assays, and biofilm elimination assays. The phage’s stability and infectivity were assessed under various pH and temperature conditions, and different *E. coli* strains. Results: Phage AUBRB02 has an incubation period of 45 min, a lysis period of 10 min, and a burst size of 30 phages/infected cell. It is stable across pH 5.0–9.0 and temperatures from 4 °C to 60 °C. Treatment with AUBRB02 significantly reduced post-formation *E. coli* biofilms, as indicated by lower OD values compared with the positive control. The whole genome sequencing revealed a genome size of 166,871 base pairs with a G + C (Guanine and Cytosine content) content of 35.47%. AUBRB02 belongs to the *Tequatrovirus* genus, sharing 93% intergenomic similarity with its closest RefSeq relative, and encodes 262 coding sequences, including 10 tRNAs. Conclusions: AUBRB02 demonstrates high infectivity and stability under diverse conditions. Its genomic features and similarity to related phages highlight its potential for phage therapy, offering promising prospects for the targeted treatment of *E. coli* infections.

## 1. Introduction

*Escherichia coli*, a Gram-negative rod from the *Enterobacteriaceae* family, is a common commensal in the gastrointestinal tracts of humans and animals. While usually harmless, it is also a prevalent opportunistic pathogen because of its ability to thrive in both aerobic and anaerobic conditions [1]. Its capacity to develop antibiotic resistance (ABR) has made it a major public health threat, particularly in healthcare settings. The emergence of antibiotic-resistant strains of *E. coli* is primarily driven by genetic exchange mechanisms such as conjugation, transduction, and transformation, as well as through acquired mutations [2].

The global issue of ABR was first observed after the discovery of penicillin, with strains such as *Staphylococcus aureus* rapidly developing resistance [3]. This has since evolved into a more significant concern because of the overuse and misuse of antibiotics in both human medicine and agricultural practices. In Lebanon and other Eastern Mediterranean countries, the prevalence of ABR is exacerbated by inadequate waste management, improper antibiotic disposal, and the widespread use of antibiotics in livestock farming [4,5]. These practices have contributed to the dissemination of resistant bacteria, not only in clinical settings but also in environmental sources such as rivers and lakes, which are vital for drinking water and agricultural purposes [6]. The emergence of multidrug-resistant (MDR) *E. coli*, including strains producing extended-spectrum beta-lactamases (ESBL), has been particularly concerning [7,8,9,10]. This necessitated the production of new antibiotics to target ABR pathogens, particularly *E. coli* (categorized as a critical pathogen by the World Health Organization in 2017) [11]. However, the discovery of new drugs is laborious and may take up to years, which makes it difficult to keep up with the emergence and spread of ABR [12]. This underscores the need for alternative strategies to treat infections caused by resistant pathogens. Among these alternatives, phages are gaining significant attention because of their specificity, self-amplification at the infection site, and lack of cross-resistance with antibiotics.

Phages are viruses that target and kill bacteria without harming host cells [13]. They are the most abundant and diverse biological entities [14], each with unique morphology and size [15]. Phages consist of a protein capsid enclosing their genetic material, which can be either DNA or RNA. They can follow various life cycles, including lytic, lysogenic, and others [16,17,18]. This study focuses on lytic phages, which use the host’s machinery to reproduce and cause bacterial cell death [19]. An ideal phage for therapy should possess several key characteristics to ensure its efficacy and safety. These include specificity to the target pathogen, the ability to proliferate at the site of infection, and a strong bactericidal effect without inducing harmful resistance in the host bacteria. Additionally, the phage should have a well-characterized genome, a stable and non-toxic profile, and the ability to be easily produced in large quantities. It is also essential that the phage does not harm beneficial microbiota and remains effective under various environmental conditions, including the host’s immune response [20]. Unlike antibiotics, phages can multiply during the bacterial-killing process, allowing for auto-dosing as they infect their bacterial targets [21,22]. Compared with antibiotics, phages have several advantages: they are self-replicating, target specific bacteria without disturbing the host’s microbiome, and are not susceptible to the same resistance mechanisms that affect antibiotics [14].

In addition to their action against planktonic bacteria, phages have shown particular promise in combating bacterial biofilms, which are known to protect bacteria from both antibiotics and the host immune response [23]. Phages have shown greater effectiveness in penetrating and disrupting bacterial biofilms than traditional antibiotics in several instances [24,25,26].

The efficacy of phages has been demonstrated in clinical trials involving animal models, highlighting their potential as a therapeutic option for MDR *E. coli* infections [27,28,29,30,31,32]. These findings highlight phages as a promising therapeutic option, especially in regions with high rates of ABR. Given the increasing burden of ABR, phage therapy represents a potential solution, particularly for infections caused by *E. coli* and other MDR pathogens.

In light of these concerns, this study aims to isolate and characterize lytic phages that exhibit activity against *E. coli* ATCC 25922 from sewage water in Ramlet El-Bayda, Lebanon. By exploring this alternative therapeutic strategy, we aim to contribute to the development of phage-based treatments that can help combat the growing problem of ABR.

## 2. Results

### 2.1. Isolation of Escherichia coli Phage

**Figure 1:**
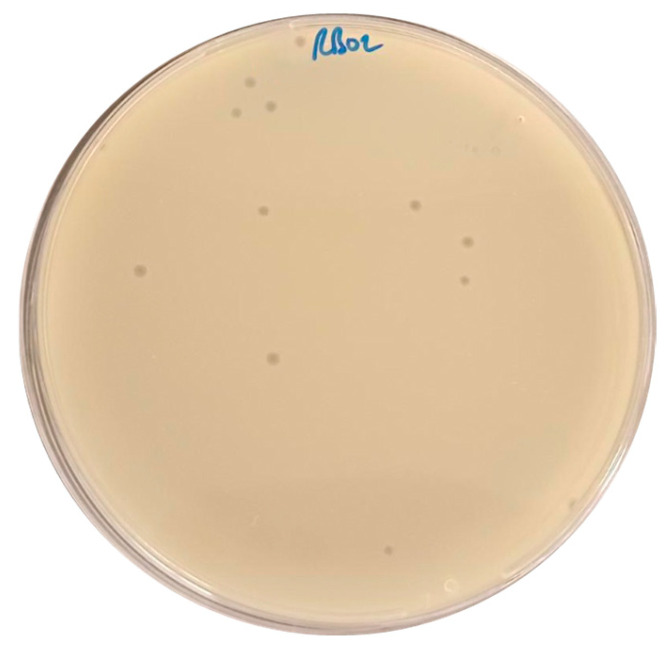
Plaque Formation of *Escherichia coli* Phage AUBRB02 on *E. coli* ATCC25922 Lawn.

AUBRB02 was isolated using *E. coli* ATCC 25922 (*E. coli* American Type Culture Collection 25922) from a sewage sample collected at Ramlet El-Bayda. A clear, circular-shaped plaque was observed on a Double-Layer Agar plate (DLA) (Figure 1) and enriched on the same host at least three times. Phage AUBRB02 formed a clear plaque, a small circular zone where the phage lysed the surrounding bacterial cells, highlighting its potential lytic activity. (In the DLA technique, a ten-fold diluted phage lysate is mixed with 0.5 McFarland *E. coli* ATCC 25922 (prepared using a densitometer) and 1 mL of Luria–Bertani broth “LB broth”, then combined with autoclaved 0.6% Luria–Bertani agar “LB agar” to form the overlay).

### 2.2. Phage Host Range Determination

**Figure 2:**
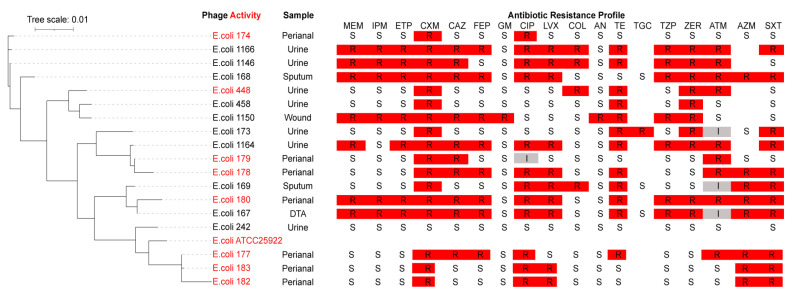
Host Range of *Escherichia coli* Phage AUBRB02 and Antibiotic Resistance Profiles of *E. coli* Strains. The phylogenetic tree on the left with a tree scale of 0.01, indicating genetic distance. Strains susceptible to *Escherichia coli* phage AUBRB02 are highlighted in red. The right section presents the ABR profiles of the *E. coli* strains against 18 antibiotics. Red squares denote resistance (R), white squares denote susceptibility (S), and grey squares denote intermediate resistance (I).

We tested the host range of phage AUBRB02 against 18 *E. coli* strains, highlighting the susceptible strains in red (Figure 2). These strains, predominantly isolated from urine, sputum, deep tracheal aspirate (DTA), and perianal samples, exhibit genetic diversity as indicated by the phylogenetic tree. The broad host range of phage AUBRB02 is demonstrated by its ability to infect genetically diverse *E. coli* strains across different clusters.

In terms of antibiotic resistance, the ABR profiles, presented in a heatmap, reveal high resistance levels, particularly to Ceftazidime (CAZ), Ciprofloxacin (CIP), Tetracycline (TE), and Trimethoprim/sulfamethoxazole (SXT). Notably, phage-susceptible strains show varying ABR levels but tend to exhibit lower antibiotic resistance compared with the non-susceptible strains. For instance, *E. coli* strains 174, 179, 178, 180, 177, and 182, while susceptible to phage AUBRB02, also demonstrate broader antibiotic susceptibility compared with the more resistant strains. Additionally, phage virulence was assessed by the Efficiency of Plating (EOP), which is the ratio of the average PFU on target bacteria to the average PFU on host bacteria. The EOP values were categorized as follows: highly virulent (EOP ≥ 0.5), moderately virulent (0.1 ≤ EOP < 0.5), slightly virulent (0.001 < EOP < 0.1), avirulent but active (EOP ≤ 0.001), or avirulent (no plaques detected) [33], as summarized in Table 1.

To further explore the host range of phage AUBRB02 beyond *E. coli*, we performed spot testing against *Acinetobacter baumannii* 1, *Klebsiella pneumoniae* 12, and *Pseudomonas aeruginosa* 44. No lytic activity was observed against these strains, indicating the specificity of AUBRB02 toward *E. coli*.

These findings underscore the genetic diversity and varying resistance profiles of the *E. coli* strains tested, as well as the broad activity of phage AUBRB02 against a range of bacterial strains.

### 2.3. Phage One-Step Growth and Adsorption Rate

**Figure 3:**
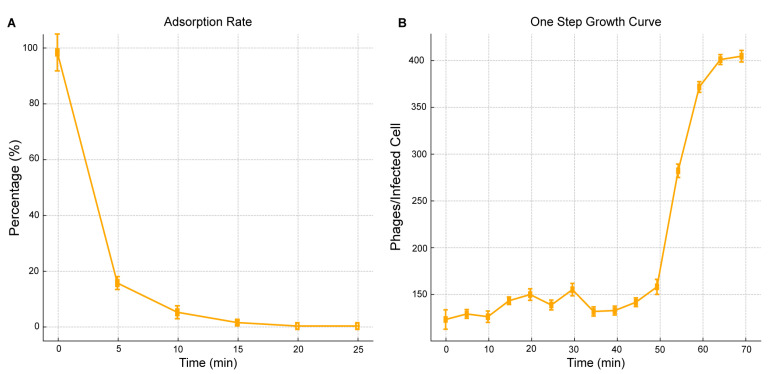
Adsorption Rate and One-Step Growth Curve of *Escherichia coli* Phage AUBRB02. (**A**) Adsorption Rate: The percentage of free phage particles not adsorbed to *E. coli* cells over 25 min is shown. (**B**) One-Step Growth Curve: The phage titer in PFU/mL over time. The shaded area represents the mean ± SD.

The adsorption rate and one-step growth curve of AUBRB02 on *E. coli* ATCC 25922 were studied to understand its host interaction. Within the first 5 min, about 80% of phages adsorbed to host cells, followed by a plateau from 15 to 25 min (Figure 3A), indicating a high affinity for the bacterial cells. The one-step growth curve revealed a 45-min latent phase before the burst phase, where the phage titer peaked at 400 plaque-forming unit/mL (PFU/mL = the number of plaques by the dilution factor and then divide the result by the volume of phage solution plated) after 60 min. The burst size was approximately 30 phages/infected cell (Burst size = PFU/mL at plateau − PFU/mL at the end of the latent period/initial number of bacterial cells (adsorption rate × bacterial concentration at time zero) = 400 × 10^7^ − 150 × 10^7^/0.83 × 10^8^ = 30 phages/infected cell), demonstrating AUBRB02’s efficiency in infecting and lysing *E. coli* ATCC 25922 (Figure 3B).

### 2.4. Bacteriolytic Activity

**Figure 4:**
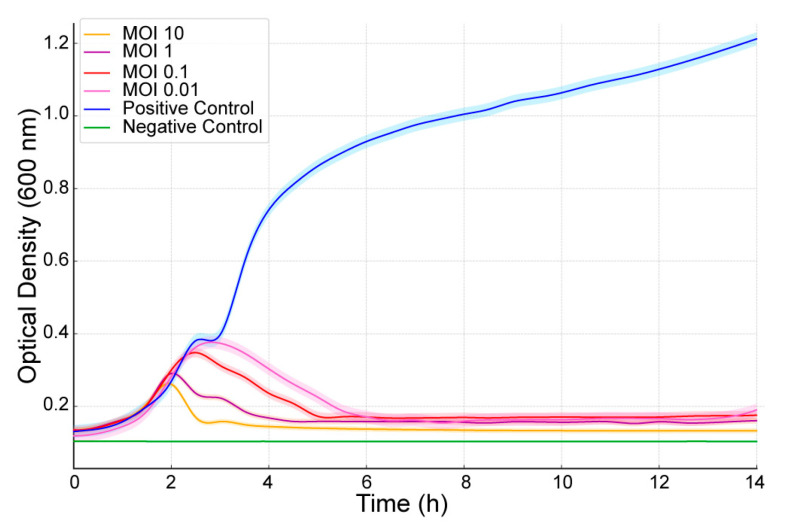
Impact of Different MOIs on the Growth of *E. coli* ATCC 25922 When Infected with *Escherichia coli* Phage AUBRB02. The graph shows the optical density at 600 nm (OD600) over time for *E. coli* ATCC 25922 treated with AUBRB02 at MOIs of 10, 1, 0.1, and 0.01 (orange, pink, red, and yellow lines). The blue line represents the positive control (phage-free bacteria), and the green line is the negative control (media only). The shaded area represents the mean ± SD.

The bacteriolytic activity of *Escherichia coli* phage AUBRB02 was evaluated at different Multiplicity of Infection (MOIs) (10, 1, 0.1, and 0.01) against *E. coli* ATCC 25922. As shown in Figure 4, OD600 was measured over time. Bacterial growth dynamics were analyzed using the Growthcurver package in R under various conditions, including bacterial controls (positive control: PC), absence of bacteria (negative control: NC), and the addition of phages at different multiplicities of infection. For the positive control (PC), robust bacterial growth was observed, with a carrying capacity (*k* = 1.005) and a high growth rate (r = 0.732). In contrast, no measurable growth was observed in the negative control (NC) or in samples with MOI values of 10, 1, 0.1, and 0.01, where these data could not be fit because of a lack of significant growth (*k* = 0, r = 0). These results suggest a strong inhibitory effect of phage treatment on bacterial growth at higher as well as at lower MOIs.

### 2.5. pH and Thermal Stability

**Figure 5:**
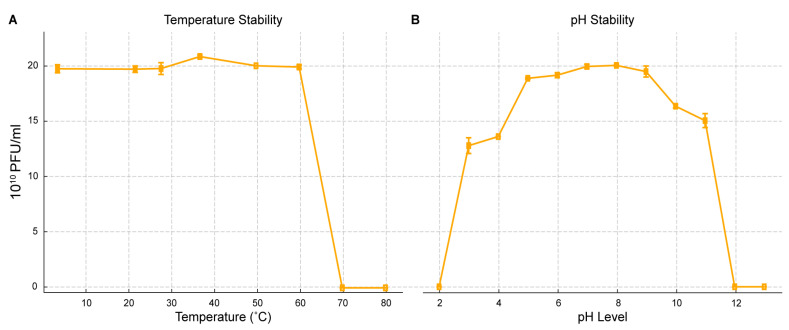
Temperature and pH Stability of *Escherichia coli* Phage AUBRB02. (**A**) The phage titer (10^10^ PFU/mL) remains stable between 10 °C and 60 °C but drops sharply above 60 °C, with complete inactivation at 80 °C. (**B**) The phage shows stability across a pH range of 4 to 10, with peak stability around pH 7 to 8. Titer decreases significantly below pH 4 and above pH 10. The shaded area represents the mean ± SD.

The stability of AUBRB02 was evaluated under different temperature and pH conditions (Figure 5), revealing its robustness. The phage maintained a high titer (~20 × 10^10^ PFU/mL) across temperatures from 10 °C to 60 °C, but the titer sharply declined above 60 °C, with complete inactivation at 80 °C (Figure 5A). Similarly, AUBRB02 remained stable across a pH range of 4 to 10, with peak stability near neutral pH (7–8). However, the titer sharply decreased below pH 4 and above pH 10, indicating sensitivity to extreme acidic or basic conditions (Figure 5B). These results demonstrate that while AUBRB02 is stable under moderate environmental conditions, it is vulnerable to extreme temperatures and pH levels, which is essential for its storage and application.

### 2.6. Biofilm Elimination Potential of AUBRB02

**Figure 6:**
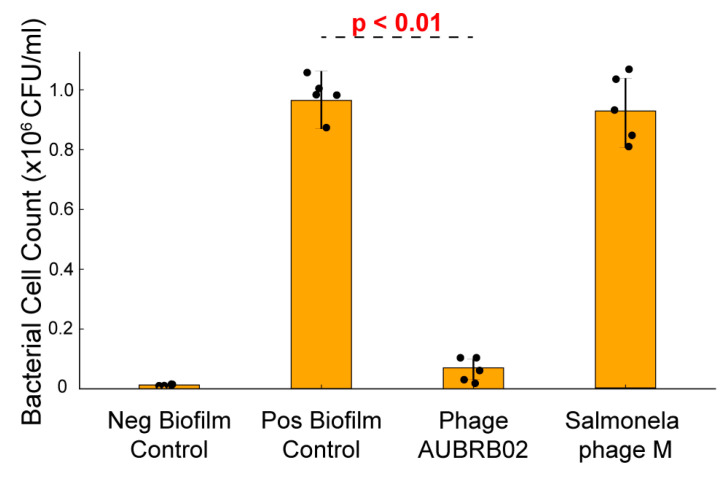
Inhibition of Biofilm Post-Formation by Phage Treatment. Bacterial cell density (expressed as ×10^6^ CFU/mL) was measured under four conditions: Negative Biofilm Control, Positive Biofilm Control, treatment with Phage AUBRB02, and treatment with Salmonella Phage M. Bars represent the mean of biological replicates, with individual data points shown as black dots and error bars indicating standard deviation. Treatment with Phage AUBRB02 resulted in a significant reduction in bacterial cell count compared to the positive biofilm control (*p* < 0.01), indicating its anti-biofilm activity.

The efficacy of AUBRB02 in reducing post-formation biofilms was evaluated. Treatment with AUBRB02 resulted in a significant reduction in biofilm, indicated by a marked decrease in bacterial cell counts (CFU/mL) by approximately 90% compared with the positive control and the control phage with no anti-biofilm activity (*Salmonella* phage M isolated specifically against *Salmonella infantis* 122,131 M). This demonstrates the phage’s effectiveness in disrupting established biofilms, underscoring its potential for targeting and reducing *E. coli* biofilm-associated infections (Figure 6).

### 2.7. Whole Genome Sequencing and Bioinformatic Analysis

**Figure 7:**
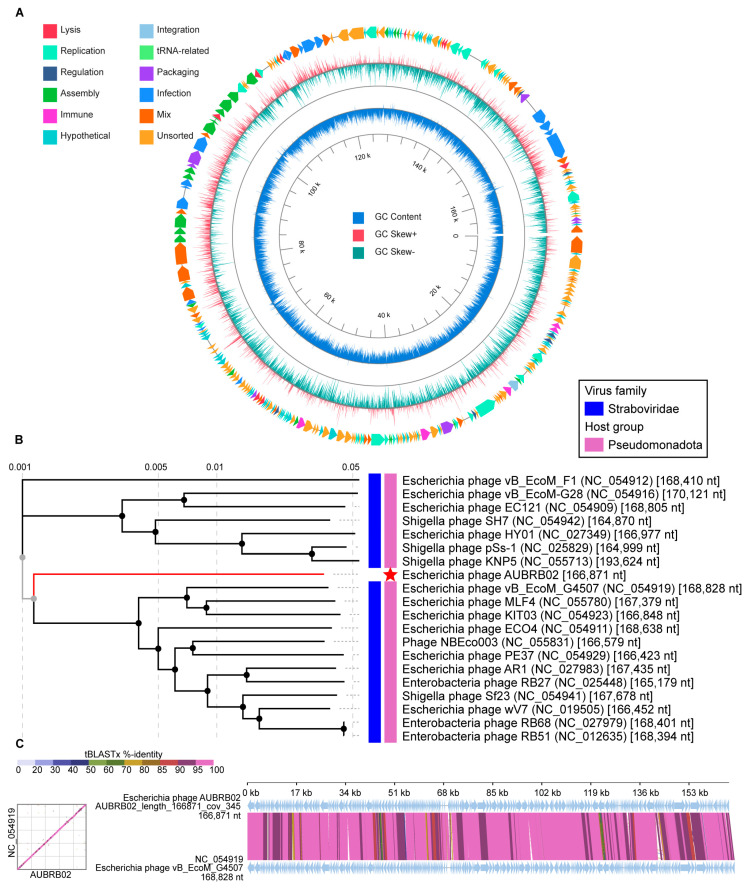
(**A**) Circular genome map showing tRNAs (light green), GC content (dark blue circle), and GC skew (red for positive, green for negative). Key structural and functional genes are annotated. (**B**) Phylogenetic tree based on tBLASTx 2.15.0, with AUBRB02 (red star) clustered within the *Straboviridae* family, showing close relationships with other *Pseudomonadota* phages. (**C**) Dot plot and synteny analysis demonstrating high sequence similarity between AUBRB02 and *Escherichia coli* phage vB_EcoM_G4507, indicating conserved genomic regions.

Phage AUBRB02 has a circular double-stranded DNA with a 166871-bp-long genome and a GC content of 35.47% (Figure 7A). The whole genome sequence of AUBRB02 was analyzed using BLASTn 2.15.0 from the NCBI non-redundant DNA database. Comparative analyses conducted demonstrated that AUBRB02 clustered with *Straboviridae* phages (Figure 7B). These phages’ alignment demonstrated that they had the same collinear genome arrangement (Figure 7C). The Virus Intergenomic Distance Calculator (VIRIDIC) analysis revealed a 93% intergenomic similarity between the compared phage genomes. Thus indicating that the genome sequence of AUBRB02 was relatively new. Functional annotation using PhageScope revealed that AUBRB02 contained 262 CDSs and 10 tRNAs (Figure 7A). The annotated proteins from the phage include a variety of functional families. Among the enzymes identified are DNA topoisomerase II, DNA primase, RNA ligase, and several helicases and polymerases. Regulatory proteins are well-represented, including transcriptional regulators such as FmdB-like and MotB-like regulators, sigma factors, and anti-sigma factors. Structural proteins encompass virion structural proteins, baseplate wedge subunits, tail fibers, and head scaffolding proteins. Numerous hypothetical proteins are present, reflecting uncharacterized or putative functions. Transport and membrane-associated proteins include RIIA and RIIB lysis inhibitors and holins. Additionally, the annotation reveals several tRNAs and other binding proteins, such as RNA polymerase binding proteins and internal virion proteins. Miscellaneous proteins include antitoxins, thioredoxins, ribonucleotide reductases, and several enzymes involved in nucleotide metabolism. Further genomic analysis using phage scope and manual inspection revealed the absence of genes related to temperate or lysogenic life cycles, as well as virulence and ABR. This indicates that AUBRB02 has a strictly lytic life cycle and lacks genes for lysogeny or antibiotic resistance virulence and ABR.

## 3. Discussion

In this study, the phage targeting *E. coli* ATCC 25922 was isolated from an untreated sewage source in Ramlet El-Bayda.

The ideal phage for phage therapy is one that targets a single bacterial species and encompasses many if not all, strains within that species. The 34% lysis effect of phage AUBRB02 suggests it is a strong candidate for therapy [34]. A broad-spectrum phage, such as AUBRB02, can act similarly to broad-spectrum antibiotics [35]. However, a major challenge is the adsorption resistance, where bacteria alter or hide their receptors, preventing phage binding [36]. Strategies to overcome this challenge include phage cocktail therapy, which involves using multiple phages targeting different receptors or bacterial strains. This could reduce the likelihood of bacterial resistance by receptor modification and expand the spectrum of phage activity [37]. Another promising approach is phage engineering, where Yehl et al. engineered the phage tail fiber by mutagenizing the receptor-binding proteins, which enhanced the phages’ ability to infect and kill bacteria that had previously developed resistance to other phages. This modification allowed the phages to overcome bacterial mechanisms that altered or hid their receptors, thereby reducing the likelihood of resistance development and maintaining efficacy against genetically diverse bacterial strains [38].

The interplay between ABR and phage susceptibility in *E. coli* is a complex area of study. Our findings demonstrate that phage-susceptible strains tend to exhibit lower ABR compared with non-susceptible strains. Research has shown that the development of phage resistance can result in increased sensitivity to antibiotics and, in some cases, a restoration of antibiotic susceptibility [39].

Research on *E. coli* phage SU57 reported a burst size of 13 phages/infected cells, which was associated with efficient bacterial killing. At an MOI of 0.14, SU57 induced a significant decrease in optical density, indicating substantial bacterial lysis. Similarly, AUBRB02, as shown in Figure 3, has a 45-min latent period and a burst size of 30 phages/infected cell, both of which are key traits for effective therapeutic phages [40,41]. This comparison underscores the superior burst size and faster replication cycle of AUBRB02, making it a highly efficient phage for therapeutic use.

The results from the phage’s one-step growth curve and bacteriolytic activity assays highlight its efficacy in lysing *E. coli* at both high and low MOIs. Phages with high burst sizes, such as AUBRB02, demonstrate efficient bacterial killing, which is critical for therapeutic applications. However, challenges may arise with phage resistance or inadequate activity in certain environments, especially in biofilm-associated infections, despite AUBRB02’s success in biofilm reduction [42].

Phage stability across a wide range of pH and temperatures is crucial for phage therapy. As shown in Figure 5, phages became inactive at 70 °C, consistent with Yamaki et al.’s findings, where high temperatures denatured the phage’s nucleic acids and proteins [43]. Additionally, AUBRB02 was inactive at pH 11, likely because of the dissociation of phage capsid proteins caused by high concentrations of hydrogen and hydroxyl ions in the solution [44].

One common bacterial defense mechanism is biofilm formation, which can trap phages upon entry and reduce the interaction between phage receptor-binding proteins and their bacterial receptors [45]. However, some phages, such as AUBRB02, have shown potential in eliminating biofilms [46,47,48]. In this study, AUBRB02 effectively eliminated *E. coli* ATCC 25922 mature biofilm, which has been used as a positive control when testing *E. coli* strains biofilm formation capabilities [49]. AUBRB02, in contrast, showed broader and more efficient disruption across the biofilm, demonstrating its potential for treating biofilm-associated infections. The annotated phage genome includes proteins, particularly glycoside hydrolases, that likely play a crucial role in breaking down the biofilm’s polysaccharide matrix, aiding in biofilm degradation [50,51]. The results demonstrate that *E. coli* biofilms can be significantly reduced through phage treatment, which holds immense potential for addressing chronic biofilm-associated infections, including those related to medical devices, chronic wounds, and cystic fibrosis-related lung infections [25]. Future studies will focus on comparing the effects of phage AUBRB02 on young and mature biofilms, as well as evaluating biofilm mass and cell viability separately. These investigations will provide a deeper understanding of phage efficacy in treating biofilm-associated infections.

The genomic analysis of *Escherichia coli* phage AUBRB02 revealed significant insights into its genetic composition and evolutionary relationships. Phylogenetic analysis (Figure 7B) placed AUBRB02 within the *Straboviridae* family, closely related to other *Pseudomonadota* phages. AUBRB02’s lack of temperate or lysogenic life cycle genes, along with the absence of virulence and ABR genes, confirms its strictly lytic nature, making it a safe candidate for therapeutic applications.

AUBRB02 is a virulent lytic phage from the *Tequatrovirus* genus, closely related to *Straboviridae* phages, and is the first *E. coli* phage isolated in Lebanon. It demonstrates high lytic activity against *E. coli* strains, characterized by a short latent period, large burst size, and rapid attachment to host cells. Importantly, AUBRB02 lacks virulence and ABR genes, making it a safe and promising candidate for therapeutic applications.

Given the growing global threat of ABR, phage therapy presents a viable alternative to treat MDR *E. coli* and other resistant pathogens. AUBRB02’s ability to effectively target both planktonic and biofilm-associated *E. coli* infections positions it as a particularly potent candidate for treating chronic infections, which are often challenging to manage with conventional antibiotics. The phage’s high burst size, rapid infection kinetics, and demonstrated biofilm degradation capacity make it an effective therapeutic tool, particularly in situations where antibiotics have failed.

To fully assess its therapeutic potential, future research should focus on clinical evaluations, including animal models and human trials. Studies have shown that phage resistance often results in increased bacterial susceptibility to antibiotics, and combining phage therapy with antibiotics or other antimicrobials can enhance overall treatment efficacy [52]. Phage-antibiotic cocktails, including combinations with AUBRB02, could mitigate resistance development, offering a strategic approach to treating resistant infections while delaying the emergence of phage resistance. This combined therapy holds great promise in managing infections caused by *E. coli* and other resistant pathogens.

## 4. Materials and Methods

### 4.1. Bacterial Strains and Growth Conditions

Bacterial strains used in this study are listed in Table 2. *E. coli*, *Salmonella infantis* 122131 (*S. infantis* 122131), *Pseudomonas aeruginosa* 44 (*P. aeruginosa* 44), *Acinetobacter baumannii* 1 (*A. baumannii* 1), and Klebsiella pneumoniae 12 (*K. pneumoniae* 12) strains were stored at −20 °C in 60% glycerol (Fisher Chemical G/0650/15, Waltham, MA, USA). Both strains were cultivated at 37 °C with shaking at 160 rpm in LB broth (LB broth Neogen Miller, Lansing, MI, USA) or plated for 18 h at 37 °C on 1.5% LB agar (LB agar Neogen Miller, Lansing, MI, USA) for *E. coli*, *P. aeruginosa*, *A. baumannii*, and *K. pneumoniae* and on *Salmonella*-*Shigella* agar for *Salmonella infantis*. The *E. coli* strains were all prepared at 0.5 McFarland for antimicrobial susceptibility testing. Antimicrobial susceptibility testing was performed by broth microdilution in a 96-well plate against 18 different antimicrobials: Meropenem (MEM), Ertapenem (ETP), Ceftazidime (CAZ), Cefepime (FEP), Zerbaxa (ZER), Aztreonam (ATM) (Merck, Darmstadt, Germany), Gentamicin (GM), Ciprofloxacin (CIP), Levofloxacin (LVX), Amikacin (AMK), Tetracycline (TE), Tigecycline (TGC), Piperacillin-Tazobactam (TZP), Azithromycin (AZM), Trimethoprim/Sulfamethoxazole (SXT), Imipenem (IPM), Cefuroxime (CXM), and Colistin (COL) (Pfizer, New York, NY, USA). Serial dilution was performed using Mueller–Hinton broth (Merck 70192, Kenilworth, NJ, USA), and the plate was incubated at 37 °C for 18–24 h. All experiments were performed in duplicates. The results were interpreted according to the CLSI M100 guidelines. Control strain *E. coli* ATCC 25922 was used in parallel to monitor minimum inhibitory concentration (MIC) results [53].

### 4.2. Sewage Sample Collection

Sewage samples were collected in 50 mL Falcon tubes from the Ramlet El-Bayda untreated sewage source in Beirut. Following 18 h sedimentation at room temperature, the samples were centrifuged at 4000× *g* for 15 min. The supernatant was subsequently filtered through a 0.22 μm disposable syringe filter to remove any residual particulates [54].

### 4.3. Phage Isolation

Sewage samples were enriched with 0.5 McFarland *E. coli* ATCC 25922 (0.5 MacFarland = 10^8^ CFU/mL) culture and supplemented with 10 mM CaCl_2_ (Calcium Chloride 10043-52-4, Sigma-Aldrich, St. Louis, MO, USA) to enhance phage growth. The enrichment process was carried out by incubating the samples for 18 h at 37 °C with shaking at 150× *g* [55]. Following incubation, the enriched samples were centrifuged at 4000× *g* for 15 min, and the resulting supernatant was filtered through a 0.22 μm syringe filter. The phage lysate was then subjected to DLA to detect phage presence against 0.5 MacFarland *E. coli* ATCC 25922 [56]. Plates were observed and scored as positive if there was a clear zone (plaque formation) over the surface of the agar plate. Plaques were picked from all positive samples, stored in SM buffer (Saline-Magnesium buffer consisting of 50 mM Tris-HCl (Merck 1185-53-1, Darmstadt, Germany) at pH 7.5, 100 mM NaCl (7647-14-5, Darmstadt, Germany) and 8 mM MgSO_4_ (Merck, 7487-88-9, Darmstadt, Germany), and counted as pfu/mL.

### 4.4. Phage Purification and Enrichment

A plaque was picked and transferred to 10 mL LB broth containing 0.5 McFarland *E. coli* ATCC 25922. After 18 h of incubation at 37 °C with shaking at 150× *g*, the mixture was centrifuged, and the supernatant was filtered using a 0.2 μm filter. The phage lysate was then diluted 10-fold in LB broth and plated using the DLA technique. This process was repeated three times until distinct plaque morphology was observed [57]. The number of plaques on the most countable plate determined the PFU/mL, which was used to calculate the MOI (MOI = Phage Concentration in “PFU/mL”/Bacterial Concentration “CFU/mL”).

### 4.5. Host Range Assay

The spot assay technique was used to determine the phage host range. Ten-fold serial dilutions of the phage in LB broth were spotted (10 µL each) onto a top agar layer (0.6% LB agar) harboring a bacterial lawn (Table 2). The plates were incubated for 18 h at 37 °C to observe any lysis zones. This experiment was performed in triplicate.

### 4.6. One-Step Growth Curve

The one-step growth curve was generated using a previous protocol from [58] with minor modifications. *E. coli* ATCC 25922 was cultured in 990 µL LB broth to a 0.5 McFarland concentration. The bacteria were mixed with 0.1 µL of phage lysate at an MOI of 0.1 (10^7^ PFU/mL/10^8^ CFU/mL) and incubated at room temperature for 5 min. The mixture was then centrifuged at 12,000× *g* for 1 min. After removing the supernatant containing free phages, the pellet was resuspended in 1 mL LB broth. This suspension was incubated at room temperature, and the phage titer was measured in triplicate every 5 min for 70 min using the DLA method.

### 4.7. Adsorption Rate Assay

To determine the time required for AUBRB02 to attach to its host *E. coli* ATCC 25922, an adsorption assay was performed following the protocol described in [59] with minor modifications. Briefly, 1 mL of phage lysate at a MOI of 0.1 (10^7^ PFU/mL/10^8^ CFU/mL) was mixed with 9 mL of 0.5 McFarland *E. coli* ATCC 25922. Samples were taken at 0, 5, 10, 15, 20, and 25 min, then centrifuged at 12,000× *g* for 3 min. The supernatants were collected and subjected to DLA assays to measure the titers of non-adsorbed phages. The DLA assay, performed in triplicate, determined the AUBRB02 titer after 18 h of incubation at 37 °C.

### 4.8. Bacteriolytic Activity Assay

Lytic activity was assessed using the protocol described in [60] against *E. coli* ATCC 25922. A 0.5 McFarland standard of *E. coli* ATCC 25922 was mixed in a 96-well plate with each phage separately at four distinct MOIs: 10 (10^9^ PFU/mL/10^8^ CFU/mL), 1 (10^8^ PFU/mL/10^8^ CFU/mL), 0.1 (10^7^ PFU/mL/10^8^ CFU/mL), and 0.01 (10^6^ PFU/mL/10^8^ CFU/mL), resulting in a final volume of 170 µL per well. The interaction between AUBRB02 and *E. coli* was performed in quadruplicate. In addition, both a negative control, consisting of LB broth only, and a positive control, containing the bacterial suspension at 0.5 McFarland with LB broth, were included. The optical density (OD) at 600 nm was recorded every 30 min over a 13-h incubation period at 37 °C with continuous shaking. Data were collected for each condition throughout the experimental time course, and bacterial growth dynamics were analyzed using the Growthcurver package in R 4.3.2. This analysis fits the observed OD measurements to a logistic growth model [61].

### 4.9. Phage pH and Thermal Stability

The stability of AUBRB02 at MOI 1 (10^8^ PFU/mL/10^8^ CFU/mL) was assessed across a range of pH levels (2–13, in increments of 1) using LB broth against 0.5 MacFarland *E. coli* ATCC 25922. Phage lysates were suspended in LB broth adjusted to the desired pH using HCl (Sigma-Aldrich, St. Louis, USA) and NaOH (Sigma-Aldrich, St. Louis, USA) and incubated for 18 h at 37 °C. The DLA assay, performed in triplicate, was used to measure the AUBRB02 PFU/mL after 18 h incubation at 37 °C. For thermal stability, phage lysates with an MOI of 1 (10^8^ PFU/mL/10^8^ CFU/mL) at pH 7 were incubated at temperatures of 4, 22, 28, 37, 50, 60, 70, and 80 °C for 2 h in a dry block thermostat. The DLA assay, conducted in triplicate, was subsequently used to determine the AUBRB02 PFU/mL after 18 h incubation at 37 °C with 0.5 MacFarland *E. coli* ATCC 25922 [62].

### 4.10. Biofilm Elimination Assay

The experiment was performed in quadruplicate using a 96-well plate to assess the effects of AUBRB02 and *Salmonella* phage M on *E. coli* ATCC 25922 biofilms. A 0.5 McFarland suspension of *E. coli* ATCC 25922 was diluted 1:100 in LB broth, and 150 µL of this dilution was added to each well designated as either a positive control (containing only the bacterial suspension) or a phage treatment. Negative control wells received 150 µL of LB broth alone. The plate was incubated for 18 h at 28 °C without shaking. Following incubation, planktonic cells were removed, and each well was washed with 150 µL of 1× PBS. AUBRB02 and *Salmonella* phage M were then introduced into the treatment wells at a multiplicity of infection (MOI) of 1 (10^8^ PFU/mL per 10^8^ CFU/mL), and the plate was incubated again at 28 °C for 5 h [63] After this second incubation, all wells were washed with 150 µL of 1× PBS, then refilled with 150 µL of LB broth. The wells were scraped, and the contents were streaked onto agar plates for colony-forming unit (CFU/mL) enumeration, following minor procedural modifications [64].

### 4.11. DNA Extraction

Phage DNA was extracted following a modified Phenol Chloroform Isoamyl alcohol protocol [62]. Phage lysate was treated with 5 µL 20 mg/mL DNase I (Roche Diagnostics, 11284932001, Basel, Switzerland) and 1 µL 10 mg/mL RNase A (Roche Diagnostics, 11119915001, Basel, Switzerland), then deactivated with 20 µL 0.5 M EDTA (Merck, E7889, Darmstadt, Germany). A volume of 2 µL of 20 mg/mL Proteinase K (Proteinase K, Hilden, Germany) and 50 µL SDS (Merck, 428018, Darmstadt, Germany) were used to digest the protein capsid. An equal volume of Phenol:Chloroform:Isoamyl alcohol (25:24:1) (Merck 136112-00-0, Darmstadt, Germany) was added, followed by centrifugation. The supernatant was treated with equal volume Chloroform:Isoamyl alcohol (24:1) (Merck C0549, Darmstadt, Germany), centrifuged again, and the supernatant was removed. DNA was precipitated using 1:10 3 M NaOAc (Merck 127-09-3, Darmstadt, Germany) and 2.5 volume 100% ethanol (Merck, 64-17-5 Darmstadt, Germany), washed with 200 µL 70% ethanol (Merck, 64-17-5 Darmstadt, Germany), and its concentration measured with a Nanodrop instrument.

### 4.12. Phage Genome Sequencing and Bioinformatics Analysis

Phage DNA libraries were prepared using the Nextera kit (Illumina, San Diego, CA, USA) and sequenced with 2 × 250 bp reads on a MiSeq system (Illumina) at the DNA Sequencing Facility of the American University of Beirut. Genome assembly and annotation of the phage AUBRB02 was performed in a Linux environment using command-line tools. Low-quality reads were filtered, and adapters were trimmed using Trimmomatic (v0.4) [65]. The filtered reads were assembled using SPAdes (v3.15.5) [66], resulting in phage genomes resolved as single contigs with an average depth exceeding 300×. The phage genome was annotated using PhageScope, an online tool specifically designed for phage genome annotation. PhageScope integrates multiple bioinformatics pipelines, including BLASTx 2.15.0searches against phage-specific databases (PHROGs, Uniprot, Pfam), conserved domain identification, and structural protein prediction. Further genomic analysis of phage AUBRB02 was conducted using Phage Scope [67], a bioinformatics tool designed to predict phage lifecycle characteristics. This tool assesses the presence of genes associated with lysogeny, virulence, and ABR. Phage taxonomy was determined using BLASTn against the NCBI database, and phylogenomic distances were scored using VIRIDIC (Virus Intergenomic Distance Calculator), with phage genomes showing ≥95% identity and coverage considered the same species [68]. A phylogenetic tree based on genome sequence similarities was constructed using VIPtree (v4.0) [69]. VIPtree employs the Genome-BLAST Distance Phylogeny (GBDP) method to calculate intergenomic distances. The GBDP method uses BLAST to compare genomes, calculating distances based on the number and length of matching fragments. Specifically, VIPtree uses BLASTn for nucleotide-level alignments. The distance matrix generated by GBDP was used to construct a phylogenetic tree with the FastME algorithm, which builds trees based on balanced minimum evolution criteria, ensuring accurate and robust phylogenetic relationships. A comparative map of AUBRB02 and the closely related phage was also created using VIPtree. The selection of vB_EcoM_G4507 was based on the fact it has a RefSeq (NC_054919.1) accession number, ensuring high-quality and curated sequence data for accurate comparative analysis. The comparison highlights key similarities and differences in genomic features.

### 4.13. Bacterial Pangenome Analysis

The bacterial genomes analyzed in this study were obtained from pathogenic bacteria identified and stored in the biorepository of the bacteriology lab at the AUB Medical Center. A total of 18 genomes were included in the pangenome analysis, conducted using Roary (v3.11.2) [70]. A pangenome phylogenetic tree was constructed from the core genome alignment generated by Roary, encompassing genes shared by all analyzed strains. This tree was visualized with iTOL (v6.0) and annotated with strain names and ABR profiles [71].

### 4.14. Statistical Analysis

Analysis and figure generation were conducted using Python (3.12.7). The one-way Analysis of Variance test was employed to determine if there were statistically significant differences in biofilm biomass between the phage-treated and control groups. A *p*-value of less than 0.05 was considered statistically significant.

All experiments were performed in three biological replicates, each with at least three technical replicates unless otherwise stated.

## 5. Raw Data Availability

Raw sequencing data for *Escherichia coli* phage AUBRB02 and the associated bacterial isolates have been deposited in publicly accessible databases. The genomic sequence of *Escherichia coli* phage AUBRB02 is available in the European Nucleotide Archive (ENA) under the accession number OY979771, with the corresponding biosample information accessible under SAMEA114786372. The project has been registered under PRJEB70649. Additionally, sequencing data for the bacterial isolates have been submitted to the Sequence Read Archive (SRA) under BioProject accession number PRJNA1224237.

## Figures and Tables

**Table 1:** AUBRB02 EOP against all *E. coli* clinical strains in comparison to *E. coli* ATCC 25922.

*E. coli* Clinical Strain	AUBRB02 EOP
174	Avirulent but active (0.000001)
1166	-
1146	-
168	-
448	High (1)
458	-
1150	-
173	-
1164	-
179	Avirulent but active (0.000001)
178	Avirulent but active (0.00001)
169	-
180	Avirulent but active (0.0000001)
167	-
242	-
177	Slight (0.002)
183	Avirulent but active (0.0001)
182	Avirulent but active (0.0001)

**Table 2:** Bacterial strains used in this study.

Bacterial Strain	Source and Other Characteristics
*E. coli* Laboratory Strains	
ATCC^®^ 25922™	Serotype 06, Biotype 01
*E. coli* Clinical Strains	
1146	Urine
1150	Wound
1164	Urine
1166	Urine
242	Urine
448	Urine
458	Urine
167	DTA
168	Sputum
169	Sputum
173	Urine
174	Perianal
177	Perianal
178	Perianal
179	Perianal
180	Perianal
182	Perianal
183	Perianal
1146	Urine
*S. infantis*	
122,131	Stool
*P. aeruginosa*	
44	Urine
*A. baumannii*	
1	Sputum
*K. pneumoniae*	
12	Urine

## Data Availability

The original contributions presented in this study are included in the article; further inquiries can be directed to the corresponding author.

## References

[1] Yoon S.H., Jeong H., Kwon S.-K., Kim J.F. (2009). Genomics, biological features, and biotechnological applications of *Escherichia coli* B: “Is B for better?!”. Systems Biology and Biotechnology of Escherichia coli.

[2] Nasrollahian S., Graham J.P., Halaji M. (2024). A review of the mechanisms that confer antibiotic resistance in pathotypes of *E. coli*. Front. Cell Infect. Microbiol..

[3] Foster T.J. (2017). Antibiotic resistance in *Staphylococcus aureus*. Current status and future prospects. FEMS Microbiol. Rev..

[4] Pormohammad A., Nasiri M.J., Azimi T. (2019). Prevalence of antibiotic resistance in *Escherichia coli* strains simultaneously isolated from humans, animals, food, and the environment: A systematic review and meta-analysis. Infect. Drug Resist..

[5] Salem S., Dahdouh E., Daoud Z. (2013). Resistance of Gram-Negative Bacilli in Lebanon. Int. Sch. Res. Not..

[6] Gaeta M., Sanfilippo G., Fraix A., Sortino G., Barcellona M., Oliveri Conti G., Fragalà M.E., Ferrante M., Purrello R., D’Urso A. (2020). Photodegradation of Antibiotics by Noncovalent Porphyrin-Functionalized TiO_2_ in Water for the Bacterial Antibiotic Resistance Risk Management. Int. J. Mol. Sci..

[7] Otrock Z.K., Oghlakian G.O., Salamoun M.M., Haddad M., Bizri A.R.N. (2004). Incidence of urinary tract infection following transrectal ultrasound guided prostate biopsy at a tertiary-care medical center in Lebanon. Infect. Control Hosp. Epidemiol..

[8] Kanafani Z.A., Kara L., Hayek S., Kanj S.S. (2003). Ventilator-associated pneumonia at a tertiary-care center in a developing country: Incidence, microbiology, and susceptibility patterns of isolated microorganisms. Infect. Control Hosp. Epidemiol..

[9] Dagher L.A., Hassan J., Kharroubi S., Jaafar H., Kassem I.I. (2021). Nationwide Assessment of Water Quality in Rivers Across Lebanon by Quantifying Fecal Indicators Densities and Profiling Antibiotic Resistance of *Escherichia coli*. Antibiotics.

[10] Moussa J., Abboud E., Tokajian S. (2021). The dissemination of antimicrobial resistance determinants in surface water sources in Lebanon. FEMS Microbiol. Ecol..

[11] Tacconelli E., Magrini N., Kahlmeter G., Singh N. (2017). Global Priority List of Antibiotic-Resistant Bacteria to Guide Research, Discovery, and Development of New Antibiotics.

[12] World Health Organization (2020). Lack of New Antibiotics Threatens Global Efforts to Contain Drug-Resistant Infections.

[13] Kasman L.M., Porter L.D. (2020). Bacteriophages. StatPearls.

[14] Ahmad T.A., Houjeiry S.E., Kanj S.S., Matar G.M., Saba E.S. (2024). From forgotten cure to modern medicine: The resurgence of bacteriophage therapy. J. Glob. Antimicrob. Resist..

[15] Dion M.B., Oechslin F., Moineau S. (2020). Phage diversity, genomics and phylogeny. Nat. Rev. Microbiol..

[16] Olszak T., Latka A., Roszniowski B., Valvano M.A., Drulis-Kawa Z. (2017). Phage life cycles behind bacterial biodiversity. Curr. Med. Chem..

[17] Wang X., Wood T.K. (2016). Cryptic prophages as targets for drug development. Drug Resist. Updat..

[18] Tavares P. (2018). The Bacteriophage Head-to-Tail Interface. Subcellular Biochemistry.

[19] Yang Z., Yin S., Li G., Wang J., Huang G., Jiang B., You B., Gong Y., Zhang C., Luo X. (2019). Global transcriptomic analysis of the interactions between phage φAbp1 and extensively drug-resistant *Acinetobacter baumannii*. mSystems.

[20] Fernández L., Gutiérrez D., García P., Rodríguez A. (2019). The perfect bacteriophage for therapeutic applications—A quick guide. Antibiotics.

[21] Carlton R.M. (1999). Phage therapy: Past history and future prospects. Arch. Immunol. Ther. Exp.-Engl. Ed..

[22] Abedon S.T., Thomas-Abedon C. (2010). Phage therapy pharmacology. Curr. Pharm. Biotechnol..

[23] Gliźniewicz M., Miłek D., Olszewska P., Czajkowski A., Serwin N., Cecerska-Heryć E., Dołęgowska B., Grygorcewicz B. (2023). Advances in bacteriophage-mediated strategies for combating polymicrobial biofilms. Front. Microbiol..

[24] Kaźmierczak N., Grygorcewicz B., Roszak M., Bochentyn B., Piechowicz L. (2022). Comparative Assessment of Bacteriophage and Antibiotic Activity Against Multidrug-Resistant *Staphylococcus aureus* Biofilms. Int. J. Mol. Sci..

[25] Liu S., Lu H., Zhang S., Shi Y., Chen Q. (2022). Phages against Pathogenic Bacterial Biofilms and Biofilm-Based Infections: A Review. Pharmaceutics.

[26] Grygiel I., Bajrak O., Wójcicki M., Krusiec K., Jończyk-Matysiak E., Górski A., Majewska J., Letkiewicz S. (2024). Comprehensive Approaches to Combatting *Acinetobacter baumannii* Biofilms: From Biofilm Structure to Phage-Based Therapies. Antibiotics.

[27] Lukman C., Yonathan C., Magdalena S., Waturangi D.E. (2020). Isolation and characterization of pathogenic *Escherichia coli* bacteriophages from chicken and beef offal. BMC Res. Notes.

[28] Inbaraj S., Angappan M., Thomas P., Kumar M., Irungbam K., Verma M.R., Viswas K.N., Abhishek, Rawat M., Chaudhuri P. (2023). Isolation and characterization of bacteriophage Ib_pec2 against shigatoxigenic *Escherichia coli*. J. Basic Microbiol..

[29] Nicolas M., Trotereau A., Culot A., Moodley A., Atterbury R., Wagemans J., Lavigne R., Velge P., Schouler C. (2023). Isolation and Characterization of a Novel Phage Collection Against Avian-Pathogenic *Escherichia coli*. Microbiol. Spectr..

[30] Vera-Mansilla J., Sánchez P., Silva-Valenzuela C.A., Molina-Quiroz R.C. (2022). Isolation and characterization of novel lytic phages infecting multidrug-resistant *Escherichia coli*. Microbiol. Spectr..

[31] Artawinata P.C., Lorraine S., Waturangi D.E. (2023). Isolation and characterization of bacteriophages from soil against food spoilage and foodborne pathogenic bacteria. Sci. Rep..

[32] Manohar P., Tamhankar A.J., Lundborg C.S., Ramesh N. (2018). Isolation, characterization and in vivo efficacy of *Escherichia* phage myPSH1131. PLoS ONE.

[33] Gibson S.B., Green S.I., Liu C.G., Salazar K.C., Clark J.R., Terwilliger A.L., Kaplan H.B., Maresso A.W., Trautner B.W., Ramig R.F. (2019). Constructing and characterizing bacteriophage libraries for phage therapy of human infections. Front. Microbiol..

[34] Hyman P. (2019). Phages for phage therapy: Isolation, characterization, and host range breadth. Pharmaceuticals.

[35] Ross A., Ward S., Hyman P. (2016). More is better: Selecting for broad host range bacteriophages. Front. Microbiol..

[36] Hyman P., Abedon S.T. (2010). Bacteriophage host range and bacterial resistance. Adv. Appl. Microbiol..

[37] Olawade D.B., Fapohunda O., Egbon E., Ebiesuwa O.A., Usman S.O., Faronbi A.O., Fidelis S.C. (2024). Phage therapy: A targeted approach to overcoming antibiotic resistance. Microb. Pathog..

[38] Yehl K., Lemire S., Yang A.C., Ando H., Mimee M., Torres M.T., de la Fuente-Nunez C., Lu T.K. (2019). Engineering Phage Host-Range and Suppressing Bacterial Resistance Through Phage Tail Fiber Mutagenesis. Cell.

[39] Chan B.K., Sistrom M., Wertz J.E., Kortright K.E., Narayan D., Turner P.E. (2016). Phage selection restores antibiotic sensitivity in MDR *Pseudomonas aeruginosa*. Sci. Rep..

[40] Bull J.J., Gill J.J. (2014). The habits of highly effective phages: Population dynamics as a framework for identifying therapeutic phages. Front. Microbiol..

[41] Duc H.M., Son H.M., Honjoh K.-i., Miyamoto T. (2018). Isolation and application of bacteriophages to reduce *Salmonella* contamination in raw chicken meat. LWT.

[42] Chegini Z., Khoshbayan A., Taati Moghadam M., Farahani I., Jazireian P., Shariati A. (2020). Bacteriophage therapy against *Pseudomonas aeruginosa* biofilms: A review. Ann. Clin. Microbiol. Antimicrob..

[43] Yamaki S., Omachi T., Kawai Y., Yamazaki K. (2014). Characterization of a novel *Morganella morganii* bacteriophage FSP1 isolated from river water. FEMS Microbiol. Lett..

[44] Feng Y.Y., Ong S.L., Hu J.Y., Tan X.L., Ng W.J. (2003). Effects of pH and temperature on the survival of coliphages MS2 and Qβ. J. Ind. Microbiol. Biotechnol..

[45] Flemming H.C., Wingender J. (2010). The biofilm matrix. Nat. Rev. Microbiol..

[46] Montso P.K., Mlambo V., Ateba C.N. (2021). Efficacy of novel phages for control of multi-drug resistant *Escherichia coli* O177 on artificially contaminated beef and their potential to disrupt biofilm formation. Food Microbiol..

[47] Pei R., Lamas-Samanamud G.R. (2014). Inhibition of biofilm formation by T7 bacteriophages producing quorum-quenching enzymes. Appl. Environ. Microbiol..

[48] Sanchez B.C., Heckmann E.R., Green S.I., Clark J.R., Kaplan H.B., Ramig R.F., Hines-Munson C., Skelton F., Trautner B.W., Maresso A.W. (2022). Development of Phage Cocktails to Treat *E. coli* Catheter-Associated Urinary Tract Infection and Associated Biofilms. Front. Microbiol..

[49] Whelan S., O’Grady M.C., Corcoran D., Finn K., Lucey B. (2020). Uropathogenic *Escherichia coli* Biofilm-Forming Capabilities Are Not Predictable from Clinical Details or from Colonial Morphology. Diseases.

[50] Redman W.K., Welch G.S., Williams A.C., Damron A.J., Northcut W.O., Rumbaugh K.P. (2021). Efficacy and safety of biofilm dispersal by glycoside hydrolases in wounds. Biofilm.

[51] Labrie S.J., Samson J.E., Moineau S. (2010). Bacteriophage resistance mechanisms. Nat. Rev. Microbiol..

[52] North O.I., Brown E.D. (2021). Phage-antibiotic combinations: A promising approach to constrain resistance evolution in bacteria. Ann. N. Y. Acad. Sci..

[53] Vanegas D., Abril-Novillo A., Khachatryan A., Jerves-Andrade L., Peñaherrera E., Cuzco N., Wilches I., Calle J., León-Tamariz F. (2021). Validation of a method of broth microdilution for the determination of antibacterial activity of essential oils. BMC Res. Notes.

[54] Abozahra R., Shlkamy D., Abdelhamid S.M. (2025). Isolation and characterization of ΦEcM-vB1 bacteriophage targeting multidrug-resistant *Escherichia coli*. BMC Res. Notes.

[55] George S., Menon K.V., Latha C., Sunil B., Sethulekshmi C., Jolly D. (2014). Isolation of *Listeria*-specific bacteriophage from three different towns in Kerala, India. Int. J. Curr. Microbiol. Appl. Sci..

[56] Svensson U., Christiansson A. (1991). Methods for phage monitoring. Bull. Int. Dairy Fed..

[57] Salifu S.P., Casey S.C., Foley S. (2013). Isolation and characterization of soilborne virulent bacteriophages infecting the pathogen *Rhodococcus equi*. J. Appl. Microbiol..

[58] Yin Y., Liu D., Yang S., Almeida A., Guo Q., Zhang Z., Deng L., Wang D. (2019). Bacteriophage potential against *Vibrio parahaemolyticus* biofilms. Food Control.

[59] Hadas H., Einav M., Fishov I., Zaritsky A. (1997). Bacteriophage T4 development depends on the physiology of its host *Escherichia coli*. Microbiology.

[60] Wang J., Zhao F., Sun H., Wang Q., Zhang C., Liu W., Zou L., Pan Q., Ren H. (2019). Isolation and characterization of the *Staphylococcus aureus* bacteriophage vB_SauS_SA2. AIMS Microbiol..

[61] Sprouffske K., Wagner A. (2016). Growthcurver: An R package for obtaining interpretable metrics from microbial growth curves. BMC Bioinform..

[62] Sada T.S., Tessema T.S. (2024). Isolation and characterization of lytic bacteriophages from various sources in Addis Ababa against antimicrobial-resistant diarrheagenic *Escherichia coli* strains and evaluation of their therapeutic potential. BMC Infect. Dis..

[63] Liu J., Gao S., Dong Y., Lu C., Liu Y. (2020). Isolation and characterization of bacteriophages against virulent *Aeromonas hydrophila*. BMC Microbiol..

[64] Bjerkan G., Witsø E., Bergh K. (2009). Sonication is superior to scraping for retrieval of bacteria in biofilm on titanium and steel surfaces in vitro. Acta Orthop..

[65] Bolger A.M., Lohse M., Usadel B. (2014). Trimmomatic: A flexible trimmer for Illumina sequence data. Bioinformatics.

[66] Bankevich A., Nurk S., Antipov D., Gurevich A.A., Dvorkin M., Kulikov A.S., Lesin V.M., Nikolenko S.I., Pham S., Prjibelski A.D. (2012). SPAdes: A new genome assembly algorithm and its applications to single-cell sequencing. J. Comput. Biol..

[67] Wang R.H., Yang S., Liu Z., Zhang Y., Wang X., Xu Z., Wang J., Li S.C. (2023). PhageScope: A well-annotated bacteriophage database with automatic analyses and visualizations. Nucleic Acids Res..

[68] Moraru C., Varsani A., Kropinski A.M. (2020). VIRIDIC—A novel tool to calculate the intergenomic similarities of prokaryote-infecting viruses. Viruses.

[69] Rohwer F., Edwards R. (2002). The Phage Proteomic Tree: A genome-based taxonomy for phage. J. Bacteriol..

[70] Page A.J., Cummins C.A., Hunt M., Wong V.K., Reuter S., Holden M.T., Fookes M., Falush D., Keane J.A., Parkhill J. (2015). Roary: Rapid large-scale prokaryote pan genome analysis. Bioinformatics.

[71] Letunic I., Bork P. (2024). Interactive Tree of Life (iTOL) v6: Recent updates to the phylogenetic tree display and annotation tool. Nucleic Acids Res..

